# Functional connectivity of the visual cortex differentiates anxiety comorbidity from episodic migraineurs without aura

**DOI:** 10.1186/s10194-021-01259-x

**Published:** 2021-05-21

**Authors:** Heng-Le Wei, Jian Li, Xi Guo, Gang-Ping Zhou, Jin-Jin Wang, Yu-Chen Chen, Yu-Sheng Yu, Xindao Yin, Junrong Li, Hong Zhang

**Affiliations:** 1 Department of Radiology, The Affiliated Jiangning Hospital with Nanjing Medical University, No.169, Hushan Road, 211100 Nanjing, Jiangsu China; 2 Department of Neurology, The Affiliated Jiangning Hospital with Nanjing Medical University, No.169, Hushan Road, 211100 Nanjing, Jiangsu China; 3 Department of Radiology, Nanjing First Hospital, Nanjing Medical University, No.68, Changle Road, 210006 Nanjing, Jiangsu Province China

**Keywords:** **M**igraine, Resting-state functional magnetic resonance imaging, Anxiety, Visual cortex

## Abstract

**Background:**

Migraine is a common neurological disease that is often accompanied by psychiatric comorbidities. However, the relationship between abnormal brain function and psychiatric comorbidities in migraine patients remains largely unclear. Therefore, the present study sought to explore the correlations between the resting-state functional deficits and psychiatric comorbidities in migraine without aura (MwoA) patients.

**Methods:**

Resting-state functional magnetic resonance images were obtained. In addition, the amplitude of low-frequency fluctuation (ALFF) and regional homogeneity (ReHo) values were obtained. Thereafter regional abnormalities in MwoA patients with and without anxiety (MwoA-A and MwoA-OA) were chosen as seeds to conduct functional connectivity (FC) analysis.

**Results:**

Compared to the healthy controls (HCs), the MwoA-A and MwoA-OA patients had abnormal ALFF and ReHo values in the right lingual gyrus (LG). They also had abnormal FC of the right LG with the ipsilateral superior frontal gyrus (SFG) and middle cingulate cortex (MCC). Additionally, the MwoA-A patients showed higher ReHo values in the left posterior intraparietal sulcus (pIPS) and abnormal FC of the right LG with ipsilateral pIPS and primary visual cortex, compared to the MwoA-OA patients. Moreover, the MwoA-OA patients showed an increase in the FC with the right posterior cingulate cortex/precuneus (PCC/PCUN), left middle frontal gyrus (MFG) and left inferior temporal gyrus (ITG) relative to the HCs. Furthermore, the ALFF values of the right LG positively were correlated with anxiety scores in MwoA-A patients. The abnormal LG-related FCs with the PCC/PCUN, MFG and ITG were negatively associated with the frequency of headaches in MwoA-OA patients.

**Conclusions:**

This study identified abnormal visual FC along with other core networks differentiating anxiety comorbidity from MwoA. This may therefore enhance the understanding of the neuropsychological basis of psychiatric comorbidities and provide novel insights that may help in the discovery of new marks or even treatment targets.

## Introduction

Migraine is an episodic disease of central nervous system that is traditionally characterized by an acute throbbing headache and heterogeneous neurological symptoms, such as photophobia, phonophobia, nausea/vomiting, mood changes and hypersensitivity to environmental stimuli. In addition, the Global Burden of Disease Study reported that migraine was the second most common neurological disease globally, leading to disability and the second-highest contributor to the burden of neurological disease, after stroke [1]. The disease therefore significantly interferes with the quality of life and increases societal burden. Notably, photophobia is the most common symptom of migraine and is one of the major criteria for the diagnosis of the disease, based on the 3rd version of the International Classification of Headache Disorders (ICHD-3) [2]. Previous studies also showed that the primary visual cortex (PVC) plays an important role in the ascending trigeminal nociceptive pathway and the descending pain modulatory system in migraine [3]. Moreover, migraineurs were reported to be more likely to experience a higher prevalence of psychiatric comorbidities such as anxiety and depression [4]. Additionally, higher intensity and frequency of headaches were shown to further aggravate the neuropsychiatric comorbidities and exacerbate the symptoms during the progression of disease [5, 6]. Furthermore, migraineurs with interictal photophobia are more susceptible to exhibit comorbidity with psychiatric disorders compared to patients without interictal photophobia [7]. However there remains much more unknown about the roles of functional information of the visual cortex in the progression of psychiatric disorders in migraine.

Since the last decade, resting-state functional magnetic resonance imaging (fMRI) has proven to be an effective, non-invasive method of examining the association between the dysfunction in the visual cortex and the pathology of neuropsychiatric disorders. Notably, the visual cortex contributes to the high-level function of cognitive control and reword processing [8]. It is also crucial for the modulation of nociception, regulation of emotions and prediction of therapeutic effect. In addition, a resting-state study showed a significant association between dysfunctions in the primary visual cortex and intensity of headache in interictal migraineurs without aura [9]. Moreover, previous studies by our research group demonstrated that many sensory networks, including the visual network, may be associated with psychiatric disorders in migraine without aura (MwoA) patients during the headache-free periods [10, 11]. Similarly, migraine patients showed significant neural activation in the lingual gyrus (LG), cuneus and precuneus after exposure to negative affective stimuli [12]. Furthermore, Yin et al. reported that the spontaneous activity pattern of the visual cortex may predict the therapeutic efficacy of acupuncture in patients suffering from migraine [13]. However, it is still unclear whether there are changes in functional characteristics of the visual cortex in episodic migraine patients with and without anxiety.

Consequently, the present study aimed to explore the association between the altered functional characteristics of the visual cortex in the interictal MwoA patients and psychiatric comorbidities. The study used amplitude of low-frequency fluctuation (ALFF) [14] and regional homogeneity (ReHo) [15] to reveal the differences in regional brain function. Specifically, ALFF measures the regional intensity of spontaneous brain activity and ReHo reflects the regional synchronization between a single voxel and its neighboring voxels. Additionally, functional connectivity (FC) analysis was used to depict differences in spatial dysfunction between remote brain areas in the subtypes of migraine. To the best of our knowledge, this was the first resting-state fMRI study based on functional characteristics to examine anxiety comorbidity in MwoA patients.

## Methods

### Participants

Forty-nine patients suffering from episodic MwoA were recruited from the neurological outpatient clinic of Nanjing Jiangning Hospital. The diagnosis of each migraineur without aura was conducted by two senior neurologists based on the ICHD-3 criteria. In addition, the inter-rater reliability between neurologists was 0.90, and a consensus on each patient’s classification was reached through discussion. In order to control the potential pharmacological effects, the study only recruited MwoA patients who had been drug-free for at least one month before being enrolled. Moreover, all the migraineurs were headache-free for at least 3 days before and after scanning. Additionally, twenty age- and gender-matched healthy controls (HCs) were selected from community volunteers. The general exclusion criteria included: (1) comorbidity with other forms of headache and nervous system diseases; (2) a history of alcohol or drug abuse; (3) pregnant or lactating women and (4) any contraindications to MRI scanning.

After diagnosis, the episodic MwoA participants underwent comprehensive tests including: the Mini-Mental State Examination (MMSE) to assess general cognitive function (all the participants MMSE scores were > 24), Visual Analogue Scale (VAS) and Headache Impact Test (HIT-6) to evaluate the severity and impact of headache and Generalized Anxiety Disorder seven-item scale (GAD-7) to measure the level of anxiety. The migraineurs were considered to have an anxiety comorbidity, when the GAD scores were > 4.Moreover, all the participants were right-handed and had at least nine years of formal education. All the study procedures were approved by the ethics committee of the Affiliated Jiangning Hospital of Nanjing Medical University.

### Acquisition of MRI images

The MRIs were conducted using a 3.0-T Philips MRI scanner (Ingenia, Netherlands) with an eight-channel head coil. All the participants were instructed to reflex and lie with their eyes closed but not fall asleep. In addition, the functional images were obtained using an echo-planar imaging sequence with the following parameters: 36 slices, repetition time (TR) = 2000ms, echo time (TE) = 30ms, 3.5-mm thickness, no gap, voxel size = 3.75 mm×3.75 mm×4.0 mm, flip angle = 90°, field of view (FOV) = 240 mm×240 mm, data matrix = 64 × 64, and 230 volumes. The functional sequence took 8 min and 8 s. Moreover, structural images were obtained using a three-dimensional turbo fast echo T1WI sequence with the following parameters: 170 slices, TR/TE = 8.1/3.7 ms, 1.0-mm thickness, gap = 0 mm, FOV = 256 mm × 256 mm, acquisition matrix = 256 × 256, and FA = 8◦. The structural sequence took 5 min and 29 s.

### Data preprocessing

Image preprocessing was performed using the Resting-State fMRI Data Analysis Toolkit plus V1.24 (RESTplus V1.24.http://restfmri.net/forum/). Preprocessing included several steps, namely: discarding the first 10 volumes, slice timing correction with the 35th slice as the reference, realignment of head motion, normalizing corrected images to the Montreal Neurological Institute space (3 × 3 × 3 mm^3^) using the diffeomorphic anatomical registration through exponentiated lie (DARTEL) algebra, regressing out the nuisance covariates (including the white matter (WM) signal, cerebrospinal fluid signal (CSF) and 6 head motion parameters), linear detrending, band-pass filtering at 0.01–0.08 Hz and spatial smoothing with a 6-mm full-width half-maximum (FWHM) Gaussian kernel. Moreover, subjects with excessive head motion in any direction (> 2 mm or 2°) were excluded from analysis.

Additionally, voxel-based morphometry (VBM) analysis was performed to segment the cerebral tissues into three tissue components, namely the gray matter (GM), WM and CSF using the Statistical Parametric Mapping (SPM12) software (https://www.fil.ion.ucl.ac.uk/spm/software/spm12/). Individual native-space GM segments were then registered to the standard Montreal Neurological Institute template using the affine registration algorithm. Thereafter, the DARTEL toolbox was applied to the GM of all participants in order to refine inter-subject registration. Subsequently, the GM tissues were modulated using a non-linear deformation approach to compare the relative GM volume adjusted for individual brain sizes. Finally, the modulated GM volumes were smoothed using an 8-mm FWHM Gaussian kernel.

### Calculation of ALFF and ReHo

Functional images without filter were transformed into the frequency domain to obtain the power spectrum using fast Fourier transform. The square root of the power spectrum was then averaged across 0.01–0.08 Hz at each voxel, and the average values were recorded as the ALFF. Following this, the ALFF of each voxel was divided by the global mean ALFF value to obtain the standardized ALFF for subsequent statistical analysis. Additionally, data without smooth was used to calculate ReHo through the Kendall’s coefficient of concordance (KCC) in order to measure the synchronization of the time series of a given voxel with its 26 nearest neighboring voxels. Moreover, individual ReHo maps were divided by the global mean ReHo value for standardization then smoothed with a Gaussian kernel of 6-mm FWHM.

### Seed-based FC analysis

Following the ReHo and ALFF analyses, the common impaired brain regions were chosen as seeds with a radius of 5 mm. Correlation analysis was then conducted between the average time course of each seed and the rest of the brain. Finally, the resulting correlation coefficients (*r*) were normalized into Z values using Fisher’s Z-transformation.

### Statistical analysis

The SPSS 25.0 software was used for statistical analyses and the level of significance was set at *p* < 0.05. The demographic characteristics of the three groups were compared using one-way analyses of variance (ANOVA)for the normally distributed continuous variables, while the Kruskal-Wallis test was employed for the non-normally distributed continuous variables. Additionally, the Chi-square test was used to make comparisons between categorical variables. Moreover, the clinical characteristics of the two groups of migraine were compared using a two-sample t-test for the normally distributed continuous variables and the Mann–Whitney test for the non-normally distributed continuous variables.

Statistical comparisons of the VBM, ALFF, ReHo and FC values were conducted through a one-way ANOVA model generated in the SPM12 software. Moreover, Post-hoc analysis was performed to identify differences between each pair of the three groups. Age, gender, and education were controlled as covariates, and the significance threshold was set at voxel-level *p* < 0.005 (Bonferroni corrected) with a 40-voxel extension. Furthermore, partial correlation analysis was performed to estimate the associations between functional impairments in the brain and the clinical characteristics (*p* < 0.05) of migraineurs, controlling for age, gender and education.

## Results

### Demographic and clinical variables

Twenty-seven MwoA with anxiety (MwoA-A) patients and twenty-two MwoA without anxiety (MwoA-OA) individuals met the inclusion criteria and were included in the study. The results in Table 1 show that there were no significant differences between the three groups with regard to age, gender or whole-brain volumes, except for education (MwoA-A vs. MwoA-OA, *p* = 0.014; MwoA-A vs. HCs, *p* = 0.540; MwoA-OA vs. HCs, *p* = 0.005). In addition, the two subgroups of migraine had no significant differences in the characteristics of the disease, including the duration of disease, frequency, duration of attacks, as well as the HIT-6 and VAS scores.

**Table 1:** Demographic and clinical variables of all participants

	MwoA-A	MwoA-OA	HCs	*F/χ*^*2*^	*p* value
Age (years)	34.41 ± 9.75	34.91 ± 12.14	33.40 ± 7.43	0.123	0.885
Sex (male/female)	1/26	6/16	3/17	5.347	0.068
Education (years)	13.44 ± 3.24	11.23 ± 2.56	14.00 ± 3.29	5.019	**0.009**
Disease duration (years)	7.11 ± 5.51	7.50 ± 6.77	/	2.930	0.824
Frequency (days/month)	4.22 ± 2.19	4.45 ± 3.00	/	0.754	0.390
Attack duration (hours)	13.22 ± 9.00	16.14 ± 12.79	/	3.590	0.355
HIT-6 score	61.26 ± 10.21	57.05 ± 7.64	/	1.733	0.116
VAS score	6.48 ± 1.42	6.45 ± 1.30	/	0.283	0.946
GAD-7 score	9.15 ± 3.30	2.59 ± 1.44	/	16.309	**< 0.001**
Gy matter (mm^3^)	621.77 ± 56.10	632.93 ± 55.92	634.71 ± 56.26	0.382	0.684
White matter (mm^3^)	484.84 ± 46.65	489.76 ± 44.96	487.40 ± 49.17	0.067	0.935
Cerebrospinal fluid (mm^3^)	208.77 ± 21.90	216.85 ± 17.50	218.56 ± 31.41	1.167	0.318
Brain parenchyma (mm^3^)	1106.61 ± 90.37	1122.69 ± 93.20	1122.11 ± 90.32	0.247	0.782

Data are presented as mean ± standard deviation. Abbreviations: *GAD-7* Generalized Anxiety Disorder seven-item scale; *HCs* healthy controls; *HIT-6* Headache Impact Test-6; *MwoA-A* migraine without aura with anxiety; *MwoA-OA* migraine without aura without anxiety; *VAS* Visual Analogue Scale

### VBM, ALFF and ReHo

**Fig. 1:**
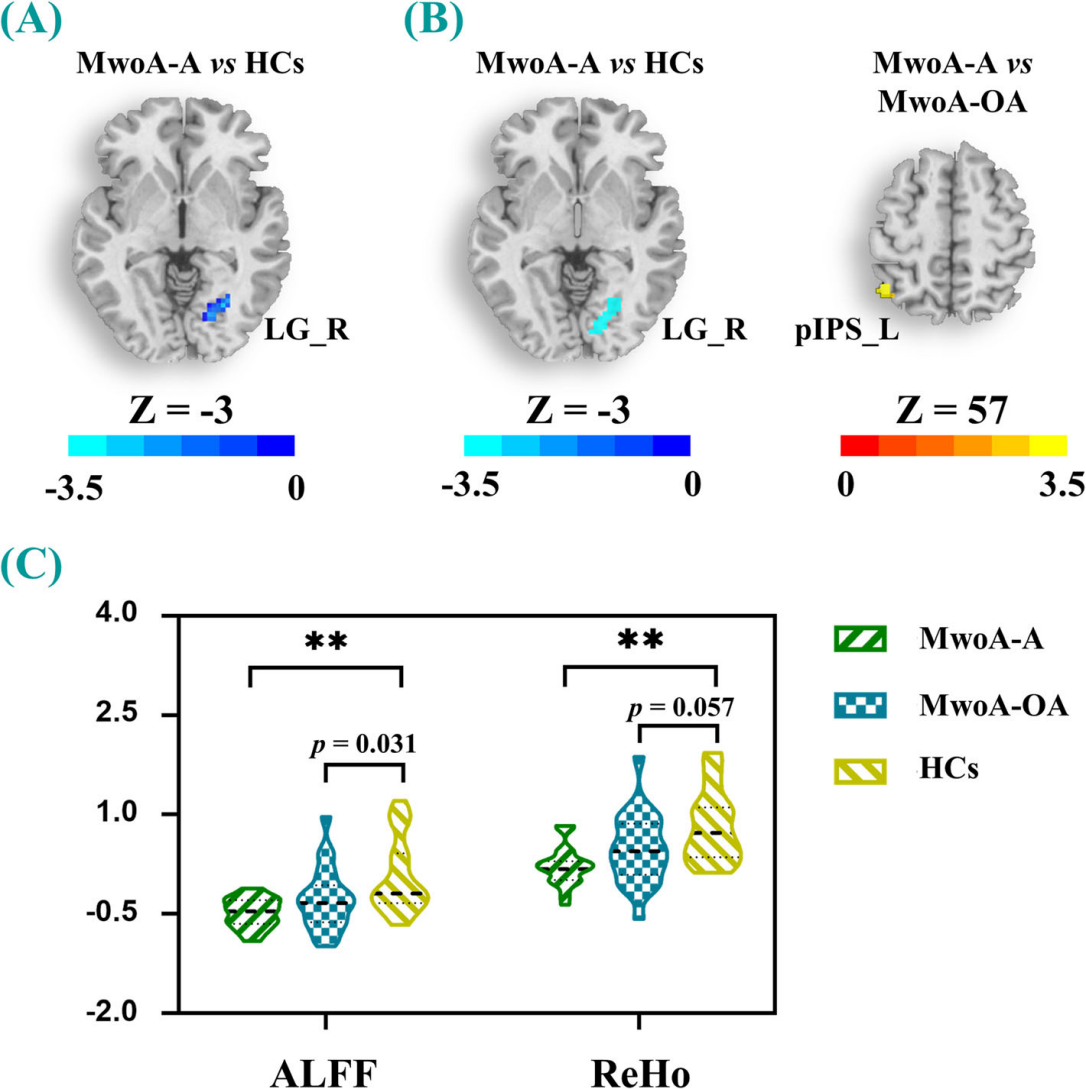
**a**-**b** Brain maps showed differences in ALFF and ReHo respectively among the three groups (MwoA-A, MwoA-OA and HCs). **c** The violin plot displayed the ALFF and ReHo values of the right LG in the three groups. ** means *p* < 0.005 (*Bonferroni* corrected). Warm and cool colors, respectively, indicate increase and decrease of ALFF and ReHo values in the right LG. Cool color represents decreased ALFF and ReHo values, and warm color represent increased ReHo values. All abbreviations are defined in the Abbreviations section

VBM analysis did not reveal any significant structural differences among the three groups. In addition, one-way ANOVA of maps revealed significantly decreased ALFF and ReHo values in the right LG of MwoA-A patients, compared to the HCs. The results also showed a decrease in the ReHo values in the left posterior intraparietal sulcus (pIPS) between MwoA-A and M-OA patients (*Bonferroni* corrected, *p* < 0.005) (Table 2; Fig. 1). However, the M-OA patients had significantly lower ALFF values (*p* = 0.031) and generally lower ReHo values (*p* = 0.057) in the right LG, compared to the HCs (Fig. 1).

**Table 2:** Regions showing differences in the ALFF and ReHo values among all groups

		MNI coordinate			K	T score
		X	Y	Z		
***ALFF***						
* MwoA-A vs. HCs*	LG_R	27	-66	-3	41	-3.6211
**ReHo**						
* MwoA-A vs. MwoA-OA*	pIPS_L	-45	-60	57	61	3.5332
* MwoA-A vs. HCs*	LG_R	24	-66	-3	63	-3.3496

Abbreviations: *ALFF* amplitude of low-frequency fluctuations; *HCs* healthy controls; *LG* lingual gyrus; *MNI* Montreal Neurological Institute; *MwoA-A* migraine without aura with anxiety; *MwoA-OA* migraine without aura without anxiety; *pIPS* posterior intraparietal sulcus; *ReHo* regional homogeneity; *L* left; *R* right. Significance threshold was set at *p* < 0.005 (*Bonferroni* corrected) with a 40-voxel extension threshold

### Seed-based FC analysis

**Fig. 2:**
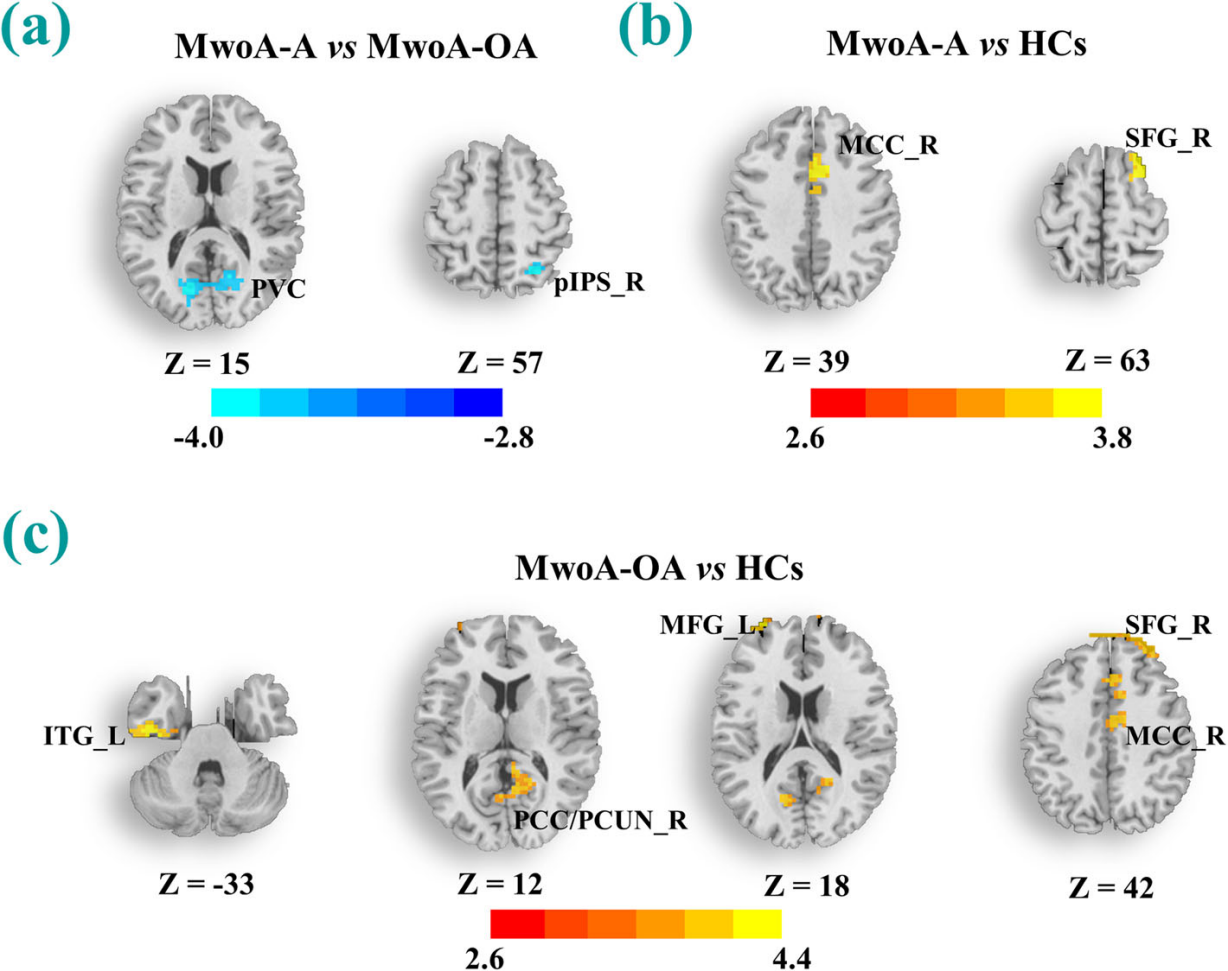
**a**-**c** The brain regions showed significant group differences in functional connectivity based on the right LG among the three groups (MwoA-A, MwoA-OA and HCs). The significance threshold was set at voxel-level *p* < 0.005 (*Bonferroni* corrected) with a 40-voxel extension. Cool color represents decreased strength of functional connectivity and warm color represent increased strength of functional connectivity. All abbreviations are defined in the Abbreviations section

The right LG was chosen as the seed to conduct seed-based FC analysis and the results are shown in Table 3; Fig. 2. Notably, the MwoA-A patients showed decreased FC of the right LG with the right pIPS and PVC, compared to the MwoA-OA counterparts. In addition, both the MwoA-A and MwoA-OA patients had a higher FC with the right superior frontal gyrus (SFG) and middle cingulate cortex (MCC), relative to the HCs. Furthermore, the MwoA-OA patients showed a higher FC with the right posterior cingulate cortex/precuneus (PCC/PCUN), left middle frontal gyrus (MFG) and left inferior temporal gyrus (ITG), relative to the HCs.

**Table 3:** Regions showing FC differences based on the right LG among all groups

	MNI coordinate			K	T score
	X	Y	Z		
*MwoA-A vs. MwoA-OA*					
pIPS_R	27	-60	57	95	-3.9292
PVC	-15	-75	15	173	-3.9957
*MwoA-A vs. HCs*					
SFG_R	24	24	63	97	3.6930
MCC_R	6	23	39	85	3.6554
*MwoA-OA vs. HCs*					
SFG_R	24	54	42	173	3.6869
MCC_R	9	-3	36	141	3.8475
PCC/PCUN_R	6	-42	12	85	3.3002
MFG_L	-30	63	18	45	4.1532
ITG_L	-45	-15	-33	110	4.4357

Abbreviations: *FC* functional connectivity; *ITG* inferior temporal gyrus; *MCC* middle cingulate cortex; *MFG* middle frontal gyrus; *PCC/PCUN* posterior cingulate cortex/precuneus; *PVC* primary visual cortex; *SFG* superior frontal gyrus. Other abbreviations are the same as in Table 2. Significance threshold was set at *p* < 0.005 (*Bonferroni* corrected) with a 40-voxel extension threshold

### Correlation analyses

**Fig. 3:**
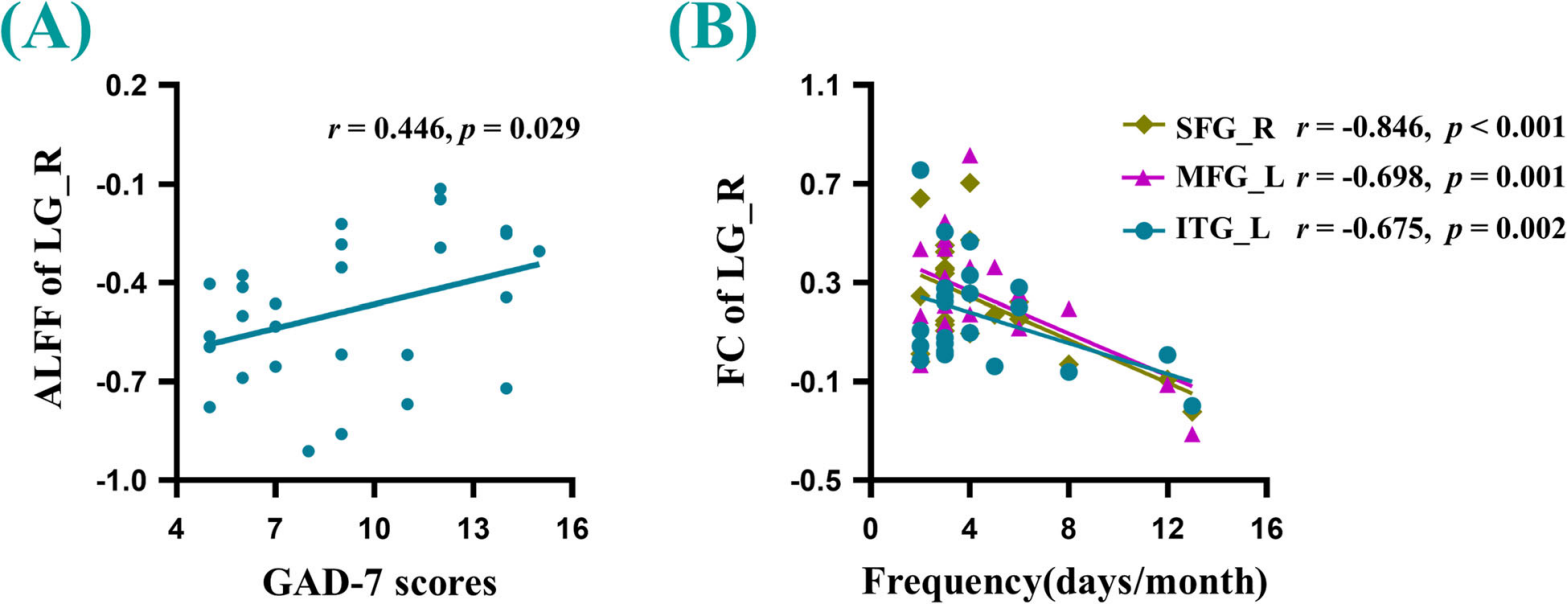
**a** There was a significant positive correlation between the ALFF values of the right LG and the GAD scores in MwoA-A patients. **b** There were significant negative correlations between the functional connectivity of the right LG with right SFG, left MFG, left ITG and frequency of migraines in MwoA-OA patients. GAD-7: Generalized Anxiety Disorder seven-item scale. All abbreviations are defined in the Abbreviations section

The findings revealed that there was a positive correlation between the ALFF values of the right LG and the GAD-7 scores (*r* = 0.446, *p* = 0.029). Additionally, the frequency of headaches in MwoA-OA patients had a significant negative correlation with visual-related FC with the right SFG (*r* = -0.846, *p <* 0.001), left MFG (*r* = -0.698, *p* = 0.001) and left ITG (*r* = -0.675, *p* = 0.002), compared to the HCs (Fig. 3). However, there was no significant association between the other forms of brain dysfunction and the other clinical parameters.

## Discussion

The present study aimed to examine differences in the FC patterns of MwoA-A patients and ascertain whether a disrupted FC may be a predictive marker of anxiety comorbidity. The results showed that the MwoA-A patients had significant regional changes within the visual cortex, which were correlated with anxiety. In addition, dysfunction in cognitive networks was associated with the frequency of headaches. These findings therefore suggested that the anxiety comorbidity is associated with a disrupted FC within many large-scale networks and may provide new insights on the pathophysiology of migraine with psychiatric disorders.

### MwoA-A vs. MwoA-OA

The MWOA-A patients in this study exhibited some unique functional changes, further supporting the distinct neural substrates of psychiatric disorders in episodic migraineurs. Specifically, the MwoA-A patients exhibited higher ReHo values in the left IPS and decreased FC between the right LG and right IPS, compared to the MwoA-OA participants. However, such abnormalities were not observed in both migraine subtypes compared to the HCs. The IPS is believed to constitute the attention network that can be activated by top-down attention in multiple sensory stimuli, including nociceptive signals [16, 17]. In addition, the findings herein corroborated with those from a previous arterial spin labeling MRI study where alterations in cerebral blood flow in the inferior parietal lobe were shown to have a significant positive correlation with an anxiety disorder in episodic migraine [18]. Moreover, Balderston et al. [19] reported that reducing IPS excitability was sufficient to reduce the physiological arousal related to anxiety, consistent with the findings in the present study. Therefore, this pattern of dysfunction in the IPS may account for the specific psychiatric features observed in migraineurs with anxiety comorbidity.

In addition, the MwoA-A patients exhibited special FC disruption at a large-scale network level within the visual cortex, compared to their MwoA-OA counterparts. Although no study has directly compared migraine patients with and without anxiety comorbidity, previous studies demonstrated that the visual cortex is associated with modulation of pain and psychiatric disorders [9, 11]. For instance, a resting-state study that combined independent component analysis and regions-of-interest FC analysis elicited that enhanced FC between the thalamus and visual cortex was associated with anxiety symptom [11]. Notably, the thalamus plays a crucial role in the trigeminovascular pathway and relays sensory information to multiple cortical networks [20]. Therefore, dysfunctions in the thalamus were shown to be responsible for disordered thinking and the anxiety network [21]. Moreover, abnormal FC between the visual cortex and amygdala was reported to be significantly correlated with the duration of migraine. It should be noted that the amygdala can serve as a core region of the mesolimbic pathway responsible for affective modulation [9]. Given the role of the visual cortex, the functional abnormalities in both the intra- and inter-visual cortex may reflect deficits of the top-down cortical inhibitory circuit and may help in distinctly characterizing migraineurs who are more prone to display overt anxiety behavior.

### MwoA-A and MwoA-OA vs. HCs

In the present study, both the MwoA-A and MwoA-OA patients exhibited similar levels of regional dysfunctions in the brain, as shown by the decreased ALFF and ReHo values in the right LG, relative to the HCs. More specifically, the neural activity of the right LG in the MwoA-OA patients was more deteriorated and was associated with anxiety. These results therefore suggest that the LG, which is a key node in the visual cortex, might be involved in nociceptive and emotional processing. To support this hypothesis, the involvement of the LG in the modulation of pain and psychiatric disorders has been confirmed through different fMRI methods [9, 22–24]. In addition to shared regional functional deficits, both the MwoA-A and MwoA-OA patients also exhibited high FC between the right LG and the ipsilateral MCC and SFG, which are core regions of the central executive network (CEN) and limbic system, respectively. These results therefore suggest that significant increase in FC between the right LG and the CEN as well as the limbic system could represent fundamental neural substrates for MwoA during the interictal period.

Notably, the CEN, including the dorsolateral prefrontal cortex and lateral posterior parietal cortex is crucial for many high-level neurocognitive functions such as cognitive performance, working memory and decision making [16, 25, 26].In addition, the MCC is an important component of the cingulate-insular pathway which gates and maintains nociceptive hypersensitivity in the absence of conditioned noxious stimuli [27] and affects the impact of headache in the migraine-free period [28]. Coupled with the aforementioned studies, the current findings suggest that the visual-related patterns of FC with the CEN and limbic system are associated with cognitive function and top-down regulation of pain. Furthermore, a pain-cognition interaction model revealed that chronic migraineurs with no exposure to painful stimuli had lower neural activity within the dorsolateral prefrontal cortex (DLPFC) and MCC compared to the HCs, but had higher neural activation in the presence of painful stimuli compared to pain-free patients [25]. Therefore, the disrupted FC patterns of the LG may direct more focus on feelings of displeasure along with recurrent headache stimuli, and further contribute to the impairment of cognitive networks and the generation of negative emotions.

### M-OA vs. HCs

Migraine and anxiety are both affected by sensitization of the central nervous system and are more sensitive to extrinsic stimuli. In addition, previous studies proved the hypothesis that migraine and psychiatric disorders may share common central nervous circuits, such as the neurolimbic network [29] and the cortico-limbic circuit [30],underlying vulnerably to nociceptive and affective stimuli [31]. Given the high comorbidity of anxiety and depression in migraine patients, it would be reasonable to consider that certain features of migraine may influence the association with anxiety. A common feature of chronic neuropathic pain is the appearance of cutaneous allodynia, which was shown to be associated with an increase in self-reported anxiety symptoms [32] and an independent risk factor of clinical anxiety [33]. Additionally, cutaneous allodynia is characterized by central hypersensitization and exacerbation in response to innocuous somatosensory stimuli [34]. Notably, some resting-state FC analysis [34, 35] showed that cutaneous allodynia was involved in the descending pain modulation pathway, including the pons, thalamus, posterior cingulate cortex, precuneus, inferior temporal cortex, middle frontal cortex and occipital cortex, in MwoA patients. These results corroborate our findings that MwoA-OA patients exhibited increased visual-related FC with the right PCC/PCUN, left MFG and left ITG compared with HCs, and make our study more stable and convincing clinically.

Moreover, the PCC/PCUN and MFG are core regions in the default mode network (DMN) and CEN, respectively, involved in self-monitoring and task processing. The DMN appears to play a dominant role during endogenous neural activity and is deactivated during the processing of exogenous cognitive tasks. Tseng [36] showed that interindividual differences in anxiety were associated with dysfunctions in the DMN especially during the encoding of painful processes. This suggested that the DMN may not be a neural substrate for the modulation of pain, but also involves a strong affective component. Additionally, Zou et al. [37] revealed that an altered DMN function could represent the relief of clinical symptoms, such as the intensity and frequency of headaches following acupuncture, consistent with previous findings by our research group [28]. Furthermore, a shift in DMN connectivity was illustrated to modulate the pain effect and evaluate the therapeutic effect. Moreover, the MFG is located in the DLPFC and plays an important role in preventing pain, through top-down inhibition pathway. Schwedt et al. [38] also revealed that enhanced pain-induced activation of DLPFC in migraineurs was positively correlated with the frequency of headaches, contrary to the results obtained in the present study. The discrepant results might be attributed to patient heterogeneity, migraine phases and study methodology. Additionally, the regional neural activity of the MFG was reported to be effective in predicting the treatment efficacy of pain and anxiety through drug therapy [39] and transcranial magnetic stimulation [40].

Furthermore, a triple network model including the DMN, CEN and salience network (SN) has been put forward to facilitate the understanding of core cognitive networks as well as psychiatric and psychological disorders, including migraine and anxiety [41]. This network model suggests that the neural activity of the DMN is opposite to the CEN when processing task-free meditation and task-related events. In this study, the visual-FC imbalance between the DMN and CEN detected in MwoA-OA patients exhibited significant correlations with the frequency of headaches, which may indicate the deficits of inhibitory function to compensate for the effects of recurrent episodes of pain. In addition, the ITG is considered to be part of the DMN [42] and has been shown to be associated with the deterioration of pain, consistent with the PCC/PCUN [43, 44]. According to the above mentioned studies, the abnormal visual-related FC patterns may account for some clinical and psychiatric symptoms in MwoA patients, such as the frequency of headaches, cutaneous allodynia and anxiety comorbidity. Moreover, medication has shown the obvious influence of regulation on the core networks (DMN and CEN) and visual network [45, 46]. Together with providing potential treatment strategies, these findings have revealed dysfunction in the integrity of migraine-relevant brain networks and may provide novel insights for the understanding of the mechanism and therapeutic strategies.

The present study has several limitations that should be considered. First, given the inadequate and subjective measurement of anxiety scores, the possibility of a classification bias cannot be ruled out. Second, the present study did not include cognitive function assessments and other psychiatric disorders. Future studies should therefore investigate the relationship between the cognitive deficits and psychiatric disorders in migraineurs. More studies are also required to assess the reciprocal effects of cognitive function and psychiatric disorders in migraine. Third, this study was restricted to the interictal MwoA patients without medication for one month, but the pharmacological effects and difference in migraine subtypes could have play a critical role in the dysfunction of the cerebral cortex and should be considered in future studies. Finally, there was no difference in the level of education between the two migraine groups, while the HCs showed a higher level of education than the other two groups. Although the main findings were obtained after controlling for the level of education, the potential confounding effects of participants’ education on the results can not be ruled out completely.

## Conclusions

In summary, the present study used data-driven analysis to show that migraine patients had overlapping regions within the visual cortex and a disrupted visual FC with the MCC and SFG, representing a common substrate for the pathophysiology of migraine. On the other hand, a disrupted visual FC within the visual cortex and with the posterior parietal lobe may be limited to the anxiety comorbidity in migraine. Additionally, the present study provides further neurobiological evidence to support the neural pathways of the triple network model acting in the sophisticated regulation of pain and anxiety only in MwoA-OA patients. Therefore, the abnormal FC patterns linking migraine and anxiety can serve as indicators of underlying psychiatric and pathophysiological neural mechanisms. The abnormal FC patterns may also guide the development of personalized pain medication for migraineurs with anxiety comorbidity.

## Data Availability

Clinical, neuroimaging and statistical data will be available upon request from any qualified investigator.

## Abbreviations

ALFF: Amplitude of low-frequency fluctuation
CEN: Central executive network
CSF: Cerebrospinal fluid
DMN: Default mode network
FC: Functional connectivity
fMRI: Functional magnetic resonance imaging
FWHM: Full-width half-maximum
GAD: General Anxiety Disorder
GM: Gray matter
HCs: Healthy controls
HIT: Headache Impact Test
ICDH: International Classification of Headache Disorders
ITG: Inferior temporal gyrus
KCC: Kendall’s coefficient of concordance
LG: Lingual gyrus
MCC: Middle cingulate cortex
MFG: Middle frontal gyrus
MMSE: Mini-Mental State Examination
MNI: Montreal Neurological Institute
MwoA-A: Migraine without aura with anxiety
MwoA-OA: Migraine without aura without anxiety
PCC/PCUN: Posterior cingulate cortex/precuneus
pIPS: Posterior intraparietal sulcus
ReHo: Regional homogeneity
SFG: Superior frontal gyrus
VAS: Visual Analogue Scale
VBM: Voxel-based morphometry
WM: White matter
